# Removal of Circulating Tumor Cells from Blood Samples of Cancer Patients Using Highly Magnetic Nanoparticles: A Translational Research Project

**DOI:** 10.3390/pharmaceutics14071397

**Published:** 2022-07-01

**Authors:** Simon Doswald, Antoine F. Herzog, Martin Zeltner, Anja Zabel, Andreas Pregernig, Martin Schläpfer, Alexander Siebenhüner, Wendelin J. Stark, Beatrice Beck-Schimmer

**Affiliations:** 1 Institute of Chemical Engineering, Department of Chemistry and Applied Biosciences, ETH Zurich, 8093 Zurich, Switzerland; simon.doswald@protonmail.com (S.D.); antoine.herzog@protonmail.com (A.F.H.); martin.zeltner@tg.ch (M.Z.); wendelin.stark@chem.ethz.ch (W.J.S.); 2 Institute of Anaesthesiology, University Hospital Zurich and University of Zurich, 8091 Zurich, Switzerland; anja.zabel@usz.ch (A.Z.); andreas.pregernig@gmail.com (A.P.); martin.schlaepfer@usz.ch (M.S.); 3 Institute of Physiology, University of Zurich, 8057 Zurich, Switzerland; 4 Department of Medical Oncology and Haematology, University of Zurich and University Hospital Zurich, 8091 Zurich, Switzerland; alexander.siebenhuener@spitaeler-sh.ch

**Keywords:** circulating tumor cells, nanoparticles, blood purification

## Abstract

The count of circulating tumor cells (CTCs) has been associated with a worse prognosis in different types of cancer. Perioperatively, CTCs detach due to mechanical forces. Diagnostic tools exist to detect and isolate CTCs, but no therapeutic technique is currently available to remove CTCs in vivo from unprocessed blood. The aim of this study was to design and test new magnetic nanoparticles to purify whole blood from CTCs. Novel magnetic carbon-coated cobalt (C/Co) nanoparticles conjugated with anti-epithelial cell adhesion molecule (EpCAM) antibodies were synthesized, and their antifouling and separation properties were determined. The newly developed C/Co nanoparticles showed excellent separation and antifouling properties. They efficiently removed tumor cells that were added to healthy subjects’ blood samples, through an anti-EpCAM antibody interaction. The nanoparticles did not interact with other blood components, such as lymphocytes or the coagulation system. In blood samples of carcinoma patients suffering from metastatic disease, on average, ≥68% of CTCs were removed. These nanoparticles could prompt the development of a blood purification technology, such as a dialysis-like device, to perioperatively remove CTCs from the blood of cancer patients in vivo and potentially improve their prognosis.

## 1. Introduction

Metastases are the leading cause of death in cancer patients [1]. They occur when tumor cells spread and colonize a distant organ [1]. Cells released from the primary tumor that travel in the blood are known as circulating tumor cells (CTCs). These may extravasate from blood vessels, infiltrate tissues, colonize niches, and eventually develop into metastases [1]. The presence and number of CTCs have been used for staging purposes as well as markers of disease progression, relapse, and overall prognosis in cancer patients. The effectiveness of such applications has been demonstrated in various types of cancer, including breast, colorectal, prostate, lung [1], urothelial [2,3], gastric, and gastroesophageal junction adenocarcinoma [4,5].

Surgery is often the primary cancer treatment, and the perioperative period is increasingly recognized as a crucial time point with potentially deleterious effects on the course of the disease [6]. Intraoperative manipulation of a tumour can release cells and is associated with a higher intravascular CTC count during and after surgery [7,8,9,10]. These CTCs might increase the risk of future metastases and disease relapse [11,12].

Current strategies to reduce the number of CTCs in the perioperative period include neoadjuvant chemo- or radiotherapy [13]. While both are beneficial for local disease control, their preventive effect on dissemination and occurrence of metastases is uncertain [14,15]. 

Diagnostic tools to detect and isolate CTCs have been developed [16,17]. These include immunocytological approaches (immunocytochemistry, CellSearch system, flow cytometry), RNA-based molecular technologies (qPCR, FISH), as well as functional assays [18]. The CellSearch system is the only detection system approved by the Food and Drug Administration (FDA) and is currently the gold standard for CTC quantification [19]. It isolates cells of epithelial origin that express the epithelial cell adhesion molecule (EpCAM) and is designed to enumerate CTCs in 7.5 mL blood samples which must be processed in order to be analyzed [20]. However, these systems are designed for diagnostic purposes. There is currently no technique available to specifically and efficiently remove CTCs in vivo from whole blood without interacting with other individual blood components. These features would allow the use of CTC removal for therapeutic purposes.

This study aimed to develop a method for efficient CTC removal from whole blood using highly magnetic nanoparticles. Carbon-coated cobalt (C/Co) nanoparticles are ferromagnetic and exhibit a magnetic saturation (158 Am^2^ kg^−1^) that is three to five times higher than commercially available (superparamagnetic) iron oxide-based nanoparticles [21]. EpCAM, as described, is expressed on cells of epithelial origin and is likely to be suitable for extracting carcinoma cells. The goal was to remove CTCs from epithelial origin from the blood of healthy subjects spiked with tumor cells as well as from blood samples of cancer patients.

**Figure 1:**
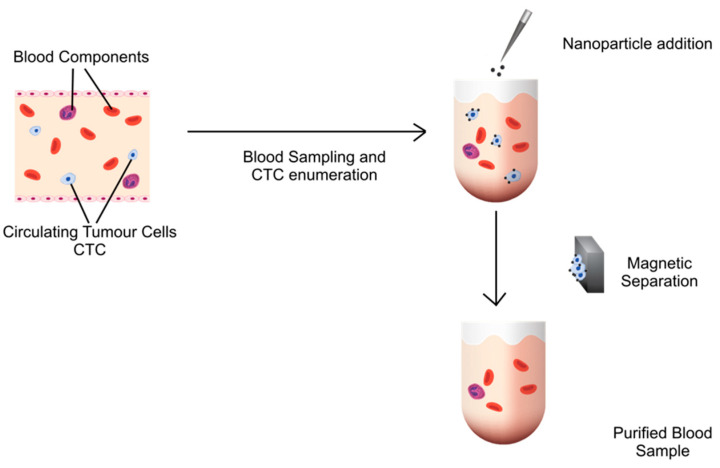
Overview of working principle in blood from cancer patients. (1) Blood from cancer patients with circulating tumour cells (CTC) (light blue) is drawn, and CTC are deterimined. (2) Nanoparticles, coated with anti-EpCAM antibodies, are added. (3) Through nanoparticles, captured tumor cells are eliminated by a strong magnet, followed by a CTC enumeration.

Such particles can lay the ground for therapeutic, in vivo whole blood purification of CTCs, such as adjuvant therapy for cancer surgery in the perioperative period which might prevent metastasis formation (Figure 1).

## 2. Methods

This study was conducted with the following steps: synthesis of the C/Co nanoparticles, characterisation of their antifouling (prevention of non-specific absorption of proteins) and separation properties, removal of CTCs from the blood of healthy subjects spiked with (cultured) tumor cells, evaluation of the effect of the nanoparticles on other blood components such as lymphocytes and the coagulation system, and removal of CTCs from blood samples of cancer patients (Figure 1). This study follows the Revised Standards for QUality Improvement Reporting Excellence (SQUIRE 2.0) publication guidelines [22].

### 2.1. Synthesis of the Nanoparticles

**Figure 2:**
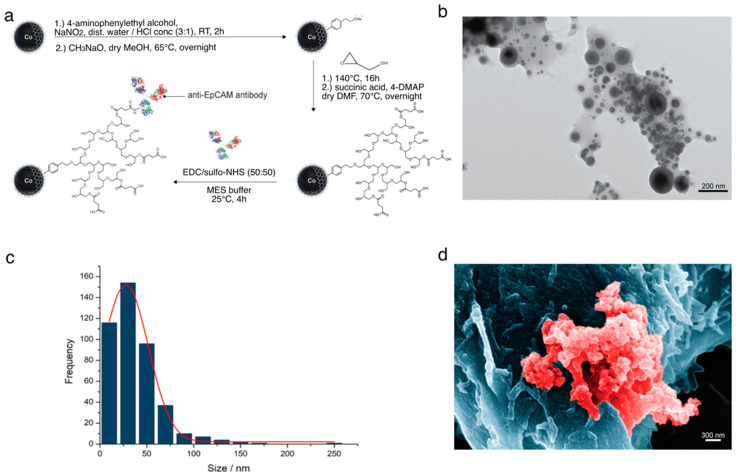
Nanoparticle synthesis and characterization. (**a**) Overview of the synthesis of antibody-functionalised Co/C nanoparticles. (**b**) Transmission electron micrographs, particle size distribution (SI). Black dots represent nanoparticles. Scale bar: 200 nm. (**c**) Graphic representation of particle size distribution. (**d**) Scanning electron micrograph of a HT-29 cancer cell after incubation with anti-EpCAM-functionalised Co/C nanoparticles. The blue area delimits a HT-29 cell, and the red area nanoparticles bound on the cell surface. Scale bar: 300 nm.

In a five-step synthesis, EpCAM-antibody functionalised magnetic nanoparticles were produced (Figure 2a). Briefly, carbon-coated cobalt nanoparticles (Nanoarmor, Katy, TX, USA), in the first step, reacted with 4-aminophenethyl alcohol (Sigma-Aldrich, Buchs, Switzerland), sodium nitrite (Sigma-Aldrich) and hydrochloric acid (VWR, Dietlikon, Switzerland) in water to obtain free hydroxy functionalities on the surface of the magnetic nanoparticles [23]. Next, the hydroxy-functionalised magnetic nanoparticles were coated with polyglycidol, a biocompatible polymer, to increase stability in the solution and reduce non-specific adsorption. This was achieved by anionic ring-opening polymerisation of glycidol (Sigma-Aldrich). For the polymerisation, the hydroxyl-functionalized magnetic nanoparticles were beforehand deprotonated to be able to act as initiator species [24,25,26]. In total, three different batches were prepared to be tested with 0, 16 and 48 polymer units. Subsequently, carboxylic acid end groups were introduced onto the polyglycidol coating of the magnetic nanoparticles with succinic anhydride (Sigma-Aldrich) and a mixture of triethylamine (Sigma-Aldrich) and 4-dimethylaminopyridine (Sigma-Aldrich) in dimethylformamide (Sigma-Aldrich) [27]. Finally, EpCAM or isotype IgG antibodies were conjugated to the free carboxylic acid end groups on the magnetic nanoparticles via EDC/NHS chemistry to obtain the antibody-magnetic nanoparticle conjugate (detailed experimental methods can be found in the Supplementary Materials).

### 2.2. Characterisation of Separation Property

The separation properties of the nanoparticles were tested using nanoparticles with different polymer units (0, 16, 48). Solutions of nanoparticles were prepared at a concentration of 2 mg mL^−1^ in phosphate-buffered saline (PBS). An amount of 4 mL of the solutions were transferred to 5 mL glass vials. The vials were placed for 10 min in an ultrasonic bath. The separation was started by placing one glass vial on each side of a permanent magnet (1.3T, Webcraft AG, Uster, Switzerland). The separation was recorded using two CMOS sensors (sensor 1: 12 MP, 1.25 µm, f/2.2; sensor 2: 12 MP, 1.0 µm, f/2.6; Xiaomi A1, Xiaomi Inc., Düsseldorf, Germany). The quantification was done using an image processing program (ImageJ, NIH, Bethesda, MD, USA).

### 2.3. Characterisation of Antifouling Property

An experiment was then conducted to test for the antifouling characteristics of the nanoparticles. Albumin (rhodamine-labelled), the most abundant plasma protein, was used. The three nanoparticle batches with the different polymer units were added to PBS (concentration of 2 mg mL^−1^). The dispersion was achieved by using an ultrasonic horn (3 × 30 s on ice, UP50H, Hielscher, Teltow, Germany). Tetramethylrhodamine-conjugated bovine serum albumin (rhodamine-BSA) was dissolved in PBS (concentration of 0.4 mg mL^−1^), and the rhodamine-BSA solution was then diluted 3 times to reach a concentration range where linear behaviour is observed for the antifouling tests. An amount of 500 µL of the rhodamine-BSA and 500 µL of each nanoparticle solution were added to an Eppendorf tube. Particles having no polymer coating were used as the negative, while milli-Q water was used as the positive control. To ensure homogeneous dispersion, the solutions were vortexed for 10 s, followed by an ultrasonication bath. The samples were shaken for 90 min at 1000 rpm at 25 °C (Thermomixer Comfort, Eppendorf, Hamburg, Germany) to ensure optimal contact between the proteins and the nanoparticle surface. The particles were separated by placing the Eppendorf tubes for 1 h in a SuperMag separator. Supernatant aliquots (5 × 100 µL) of each sample were transferred to a 96-well plate before fluorescence was measured (ex: 540 nm, em: 620 nm; Spark 10 M; Tecan, Männedorf, Switzerland).

### 2.4. Cell Line Experiments

To obtain human tumor cells for the spiking experiments, the EpCAM-expressing human colon cancer cell line HT-29 (HTB38, ATCC) was cultured in RPMI 1640 medium, supplemented with glutamax (Gibco, Zug, Switzerland), 10% fetal bovine serum (FBS, Gibco) and a mixture of penicillin/streptomycin (final concentration 100 U mL^−1^/100 µg mL^−1^, Gibco). The cells were incubated at 37 °C in 5% CO_2_. Once cells reached confluence, they were detached with accutase (Gibco) and resuspended in blood at 5 × 10^5^ cells mL^−1^. The cell membranes of the HT-29 cells were stained with the PKH26GL labelling kit (Sigma-Aldrich) before they were added to whole blood for the experiments. Cell passages between 5 and 40 were chosen for these experiments.

### 2.5. Ethics Ex Vivo Part, Healthy Volunteers

Ethical approval for blood sampling from healthy volunteers (Ethical Committee N° 2012-0274) was provided by the local Ethical Committee (Kantonale Ethikkommission Zurich, Zurich, Switzerland), Chairperson Prof. Dr. Peter Meier-Abt on 14.08.2012.

### 2.6. Removal of CTCs from the Blood from Healthy Subjects Spiked with Tumor Cells

After informed consent, blood samples up to 20 mL were drawn from healthy subjects into heparin tubes (BD Biosciences, Allschwil, Switzerland). The blood was then spiked with HT-29 cells to a final concentration of 5 × 10^5^ cells mL^−1^. 

Eppendorf tubes containing a solution with anti-EpCAM nanoparticles (2.38 mg mL^−1^ in PBS) were sonicated (5 × 1 min with 1 min breaks in between) in an ultrasonication bath in ice-cold water. In addition, a control solution with nanoparticles, conjugated with an IgG isotype control antibody (2.38 mg mL^−1^ in PBS), was prepared the same way. Once sonicated, 25 µL of either solution was added to 475 µL of blood containing tumor cells (final concentration of nanoparticles: 0.119 mg mL^−1^). A second control was established with only 25 µL PBS as carrier solution without nanoparticles. The samples were then incubated for 2 min on an orbital shaker (16 mot min^−1^, WS 10, Edmund Bühler, Bodelshausen, Germany) before being passed through a magnet column system (MS columns, MACS, Miltenyi Biotec, Bergisch Galdbach, Germany). Each column was washed twice with 500 µL of PBS, which was also collected as filtrate.

### 2.7. Analysis of Samples Using Fluorescence-Activated Cell Sorting (FACS)

The filtrates were then prepared for flow cytometry analysis based on fluorescence-activated cell sorting (FACS). Red blood cells were lysed by adding 10 mL of red blood cells lysis buffer (Biolegend, San Diego, USA), and samples were centrifuged for 10 min at 400× *g* (centrifuge 5810R, Eppendorf). The supernatant was discarded, and the pellet was resuspended in 200 µL PBS. The cells were fixed with 200 µL of a 4% formalin solution. An amount of 25 µL of counting beads solution (concentration of 5.2 × 104 counting beads per 50 µL, CountBright Absolute, Life Technologies, Zug, Switzerland) was added to each sample before analysis using a flow cytometer (BD Canto II, BD Biosciences). The acquisition was carried out with the BD FACSDiva Software (BD Biosciences). Forward, side scatter area, and signal height were recorded. Circulating tumor cells were detected using the forward scatter area vs. the PE-A. Fluorescence-activated cell sorting data were processed using FlowJo V10.0.8, Ashland, OR, USA.

### 2.8. Evaluation of a Possible Effect of Nanoparticles on Blood Cells

We investigated if the nanoparticles interact with other blood cells or components such as lymphocytes. Blood samples from healthy volunteers were exposed to anti-EpCAM nanoparticles or to PBS without nanoparticles, as described above. The samples were run over the magnetic column system. Lymphocytes were labelled by incubating the filtrates with human anti-CD19 APC-conjugated (SJ25-C1, ThermoFisher, Waltham, MA, USA; final concentration: 0.2 mg mL^−1^) and human anti-CD3d Alexa Fluor 48-conjugated (AB, ThermoFisher; final concentration: 0.2 mg mL^−1^) antibodies for 30 min at 4 °C. Red blood cells were then lysed, and samples were prepared for flow cytometry analysis, as mentioned above. Forward and side scatter area, as well as signal height, were recorded. Lymphocytes were detected using either side scatter area or forward scatter area and APC-A vs. Alexa Fluor 488-A. FACS data were processed using FlowJo V10.0.8.

### 2.9. Evaluation of a Possible Effect of Nanoparticles on the Coagulation System

To assess the effects of nanoparticles on the coagulation system, blood samples were incubated with anti-EpCAM or with IgG isotype particles, solved in PBS as described. After letting the samples run over the magnetic column, rotational thromboelastometry (ROTEM), a clinically well-established viscoelastic method to test haemostasis in whole blood as a reliable point-of-care tool, was performed. ROTEM provides detailed information about clot formation such as clotting time (CT), clot formation time (CFT) or maximum clot firmness (MCF), reflecting the strength of the clot via two activation pathways (EXTEM: activation via tissue factor; INTEM: contact activation).

### 2.10. Ethics Ex Vivo Part Cancer Patients

Ethical approval for blood sampling from cancer patients (Ethical Committee N° 2016-01140) was provided by the local Ethical Committee (Kantonale Ethikkommission Zurich, Zurich, Switzerland), Chairperson Prof. Dr. Erich Russi on 24.11.2016. The study is registered as part of a multi-step project on clinicaltrials.gov, study identifier: NCT04290923.

### 2.11. Removal of CTCs from Blood Samples of Cancer Patients

Upon obtaining informed consent, blood samples from cancer patients suffering from metastatic disease were drawn and considered for eligibility for the project. The samples were collected in CellSave (Menarini-Silicon Biosystems, Castel Maggiore, Italy) or EDTA tubes. The presence of CTCs in the samples was established using the CellSearch system, with a cut-off value of 5 cells per 7.5 mL to proceed with the nanoparticle-based CTC removal. Eligible blood samples were treated with either the anti-EpCAM nanoparticle solution (2.38 mg mL^−1^ in PBS) or with PBS as described. As analysis of such low CTC counts is not feasible with the FACS method, the filtrates were determined using the CellSearch system. The detection limit was one cell in 7.5 mL of blood. Results were evaluated by trained experts with CellSpotter.

### 2.12. Data presentation and Statistical Analyses

**Figure 3:**
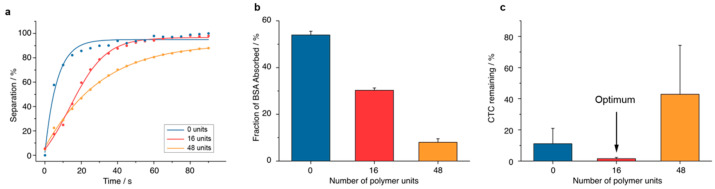
Nanoparticle optimization. (**a**) Separability: The graph depicts a representative of several experiments performed with different batches. (**b**) Antifouling: the fraction of bovine serum albumin (BSA) absorbed was determined in particles with a different number of polymer units. (**c**) Efficiency: CTC-absorption was determined using nanoparticles with a different polymer unit.

**Figure 4:**
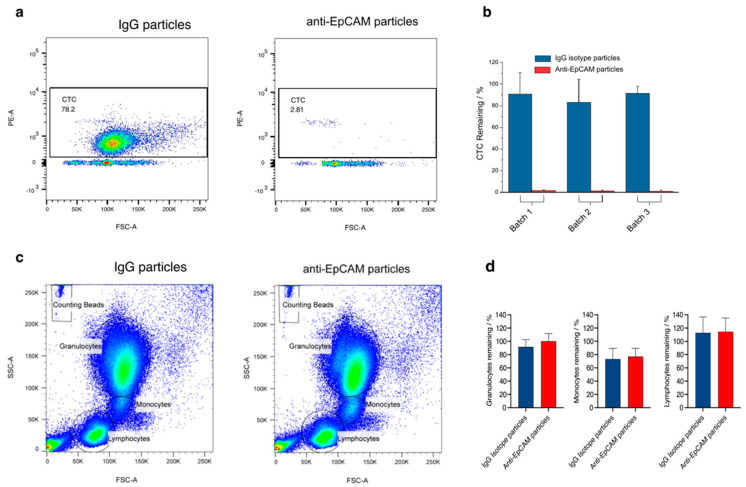
Removal of CTCs. (**a**) Flow cytometry data of filtrates obtained after removal of CTCs from healthy donors’ blood spiked with tumor cells using IgG isotype and anti-EpCAM-coated nanoparticles. The gate indicates the region where the spiked HT-29 cells appear. (**b**) Reproducibility of the experiment, which was repeated three times with three different optimized anti-EpCAM nanoparticles batches in comparison to IgG istotype nanoparticles. (**c**) Flow cytometry results after treatment of blood with anti-EpCAM-functionalized nanoparticles focusing on granulocytes, monocytes and lymphocytes. (**d**) Percentage of remaining granulocytes, monocytes and lymphocytes after IgG isotope or anti-EpCAM particle treatment.

Data of Figure 3b,c and Figure 4b are presented as mean with standard deviation (SD). The two groups were compared using a two-tailed Student’s *t*-test. Statistical significance was defined at the 5% level (*p* < 0.05). All statistical analyses were performed using the GraphPad Prism for Mac software (version 8.1.2, GraphPad Inc., La Jolla, CA, USA).

## 3. Results

### 3.1. Optimal Nanoparticle Characteristics

#### 3.1.1. Synthesis and Separation Capability

Nanoparticles are usually engineered to be as stable as possible. However, this often results in poor separability and performance in filtration applications. This was demonstrated by preliminary experiments with recently developed nanoparticles with poly(3-sulfopropyl methacrylate potassium salt) (pSPM) as polymer [28]. Therefore, polyglycidol was selected as a polymer for the nanoparticles in this study. The electrosteric repulsions are much lower than for pSPM, providing adequate separability while maintaining antifouling properties. In addition, polyglycidol is biologically inert and approved by the FDA [29,30,31].

The nanoparticle synthesis and size are shown in Figure 2a,b. Assuming that colloidal stability is directly dependent on the extent of the polymer coating, three nanoparticle batches with different polymer lengths were manufactured with no polymer spacer (‘0’), 16 or 48 polymer units. The particles were then conjugated with anti-EpCAM antibodies: Figure 2c depicts the nanoparticles, while Figure 2d shows aggregates of these nanoparticles on HT-29 tumor cells, both analyses performed by transmission and scanning electron microscopy, respectively.

For qualitative information about the relative magnetic separability of the three batches, the different particles were dispersed in PBS and separated from the solution using a permanent magnet. The separation speed was recorded with a camera and quantitatively analyzed with image processing software. Increasing the amount of functionalized polyglycidol (from 0–48) units increased separation by a magnet from seconds to several minutes (Figure 3a).

#### 3.1.2. Antifouling Properties

To assess the antifouling properties of the nanoparticles with the different polymer groups, the nanoparticles were exposed to rhodamine-labelled albumin, the most abundant plasma protein [32]. After incubation and magnetic collection of the nanoparticles, the fluorescence of the supernatant was measured, and protein adsorption was calculated using a standard curve. A higher degree of polymerization accounted for better antifouling properties. Particles functionalized without spacer demonstrated a 6.8-fold higher bovine serum albumin (BSA) adsorption than particles with 48 polyglycidol units (Figure 3b).

#### 3.1.3. CTC Removal Efficiency 

The CTC removal efficiency of the three types of nanoparticles was assessed in blood from ten healthy donors spiked with HT-29 tumor cells. The nanoparticles that provided the highest efficiency for CTC removal were functionalized with an average of 16 units per polyglycidol chain (Figure 3c).

Based on the results of the separation capability, the antifouling properties and the efficiency, nanoparticles with an average of 16 polyglycidol units were considered the most promising material. The following experiments were all performed with this batch. 

#### 3.1.4. Specificity of CTC Removal

Removal of CTCs by the functionalized nanoparticles should result from an EpCAM epitope-antibody interaction and not from the non-specific binding of tumor cells to the surface of the nanoparticles. The experiment was repeated with nanoparticles conjugated with the IgG control antibody to confirm specificity. The removal efficiency was then compared with anti-EpCAM nanoparticles. With IgG isotype nanoparticles (control), between 83% and 91.4% of the cancer cells remained in the blood samples, with anti-EpCAM nanoparticles only 0.8 to 1.5% remained (Figure 4a,b).

#### 3.1.5. Testing of Possible Adverse Effects

While the specific removal of cancer cells is desired, non-specific interaction with other blood cells should be avoided. To exclude unintentional removal of B- or T-lymphocytes, their cell count was determined by flow cytometry after exposure to anti-EpCAM nanoparticles (or IgG particles as control) and purification using the magnetic column system. The B- and T-lymphocyte count was comparable, whether or not blood samples were treated with anti-EpCAM nanoparticles (*p* = 0.6 and *p* = 0.5, respectively) (Figure 4c and Table 1). Moreover, the cells in the gates for granulocytes, lymphocytes, and monocytes were evaluated. No difference was observed between IgG vs. Anti-EpCAM nanoparticle treatment (*p* = 0.4, *p* = 0.8 and *p* = 0.9, respectively, Figure 4d).

Additionally, a potential interaction of the nanoparticles with the coagulation system using ROTEM was assessed. For blood samples treated with anti-EpCAM nanoparticles, all EXTEM and INTEM ROTEM data were within the reference values (Table 2). EXTEM and INTEM assess the coagulation cascade induced by the tissue factor and contact activation, respectively (Table 2) [33].

### 3.2. From the CTC In Vitro Model to Blood from Cancer Patients

Finally, nanoparticles were tested with blood samples from cancer patients. Between October 2017 and March 2018, blood samples of 41 patients with metastatic cancer were assessed for eligibility. Of 41 samples, 8 met the cut-off of five cells/7.5 mL to proceed with the CTC removal experiment. Three samples were from prostate, three from colon cancer, and two from pancreatic cancer patients. Sample treatment was identical to the treatment of healthy volunteers’ blood samples (see above), with the only difference in the sample volume. The samples were 7.5 mL rather than 0.5 mL, all volumes were scaled up by a factor of 15. 

A measurement error was observed in two samples of colon cancer patients, and the results could not be interpreted. For the remaining six samples, an average of ≥68% of CTCs were removed with a single passage through a magnet column. The detailed results are shown in Table 3.

## 4. Discussion

For this study, Co-C nanoparticles were covalently functionalised with a polyglycidol coating and anti-EpCAM antibodies to purify blood samples from CTCs. These nanoparticles exhibited a high degree of magnetisation while conserving good separability and the necessary antifouling properties for this application. They specifically interacted with CTCs through their anti-EpCAM antibody, while other blood cells were not involved in the removal process. Additionally, no influence on the coagulation system was observed. The manufacturing process presented little variability between batches, and CTC removal efficacy was consistent. This allowed efficient CTC removal from the blood of healthy subjects and blood samples of cancer patients with metastatic disease.

The use of magnetic nanoparticles for medical purposes has developed dramatically in recent years. The possibility of manufacturing the desired particle size and coating with biological or pharmaceutical molecules provides diverse applications, ranging from drug delivery to imaging enhancement [34]. One of the most notable applications of magnetic nanoparticles has been separating and isolating specific cells, notably CTCs. This has aided in the diagnosis and in the staging of cancer patients and monitoring therapeutic response. Isolation of CTCs is not without challenges, partly due to their very low concentration in the blood (from 1–10 cells per 10 mL) [18]. Currently, only one system for diagnostic CTC isolation exists. This implies that their application is restricted to low volumes of blood. Moreover, for diagnostic purposes, interactions with other blood components are irrelevant. Whereas for therapeutic purposes, time-efficient purification of large volumes of blood without preprocessing is essential, and it is crucial to avoid any side effects on other blood components. 

A strength of our study is that it encompasses many steps, from manufacturing to clinical testing of blood samples of cancer patients. The advantages provided by our nanoparticles are notably faster clearance without compromising the separability or antifouling characteristics. Additionally, no adverse effects were found, such as non-specific blood cell removal or interaction with the coagulation system.

This study also has limitations. As a proof-of-concept study, the sample size for the CTC removal experiment from cancer patients’ blood was small. However, the effect was consistent, even though patients suffered from varying cancer types and had unequal CTC concentrations. A larger sample size will be required to confirm a consistent effect across different malignancies or highlight an improved efficacy for some cancer subtypes. Between 37% and 99% of CTCs (mean value: 70%) were removed from cancer patients’ blood with one addition of nanoparticles. The clinical implications of such a value are still unknown. To increase blood purification efficiency, repeating the elimination process might be necessary. An optimized particle production with a smaller size variance might additionally improve purification. Furthermore, nearly complete removal of the particles has to be ensured since toxic effects through cobalt-iron leaching cannot be excluded. However, bioreactivity of cobalt is mainly observed in acidic pH [35]. Additionally, the contact time between nanoparticles and blood is relatively short (only minutes). 

Another limitation is that only one cell line was used, namely the HT-29 colon cancer cells. However, this study aimed to focus on the removal of EpCAM-expression cancer cells, independent of the entity of the tumor cell. This is exactly the mechanism the CellSearch system uses, allowing a broad application. 

Finally, one must realise that tumor cells expressing EpCAM may undergo epithelial mesenchymal transformation with a reduction of EpCAM expression [36]. In an advanced stage of this process, such cells will no longer be detected by EpCAM antibodies; therefore, elimination will be reduced. 

While only a tiny fraction of CTCs colonize a distant organ, CTCs are a known source of metastases. A high CTC count has been associated with an elevated risk of metastases or a worse prognosis in many types of cancer [37]. In addition, surgery, often the treatment of choice, has been linked to an increase in CTC counts perioperatively [14]. The nanoparticles presented in this article could provide the basis for developing a whole blood filtration technology for CTC removal. Extracorporeal blood purification is an attractive method of removing undesirable non-cellular and cellular compounds from the blood. Some well-known blood purification methods include haemodialysis or the use of filters. While these systems are suitable for removing small molecules, they are not optimised for larger compounds [38]. 

Using magnetic nanoparticles allows a reliable and specific removal of small and large compounds. To date, sepsis has been the main focus of novel blood purification systems with techniques such as CytoSorb [39]. However, no blood purification device exists for CTCs. We envision a device akin to a dialysis machine, which could purify the blood of CTCs extracorporeally in vivo using the nanoparticles presented in this study. Such a device might be used at any time point in the perioperative timeframe to limit the dispersion of CTCs. Biosafety of the nanoparticles is crucial for such an application. While interference with lymphocytes and the coagulation system could be excluded, a potential concern could be the nanoparticles re-entering the patient’s body after filtration. However, our group co-developed a method to detect nanoparticles in blood down to sub-picomolar concentrations [33]. Using such a system combined with a blood purification device would prevent nanoparticle-contaminated blood from re-entering the patient’s circulation [40]. Future studies are required to confirm the biosafety of the nanoparticles and the feasibility of using them with a blood purification device which would require another significant up-scale of our experimental setup. Finally, such studies would also have to investigate if reducing the CTC count perioperatively improves prognosis and reduces the occurrence of metastases.

In conclusion, this study presents the development of highly magnetic C/Co nanoparticles conjugated with anti-EpCAM antibodies to separate CTCs from whole blood. The advantage of these nanoparticles lies in their scalability and ease of synthesis. As shown in the experiments, these magnetic nanoparticles can remove CTCs in specific cases without significantly interfering with other blood cells or components. Therefore, such nanoparticles may lay the ground for developing an adaptable blood purification technology, similar to dialysis, to perioperatively remove CTCs from the blood of cancer patients and potentially improve their prognosis.

## Figures and Tables

**Table 1:** B- and T-lymphocyte count in three different blood samples after treatment with anti-EpCAM-coated magnetic nanoparticles (or IgG isotype particles as control). The number of B-lymphocytes and T-lymphocytes remains similar after IgG vs. Anti-EpCAM nanoparticle treatment (*p* = 0.6 and *p* = 0.5). *n* = number.

Particles	B-Lymphocytes (*n* Cells per 10^6^ Counting Beads)			T-Lymphocytes (*n* Cells per 10^6^ Counting Beads)		
	Test 1	Test 2	Test 3	Test 1	Test 2	Test 3
IgG	3529	2936	1641	34888	27673	20943
Anti-EpCAM	4233	2927	1447	40493	28682	19231

**Table 2:** ROTEM measurements in blood from healthy subjects. The measurements were made in blood supplemented with either anti-EpCAM nanoparticles or IgG isotype particles (control). Clotting time (CT), clot formation time (CFT) and maximum clot firmness (MCF) for the EXTEM channel (activation via tissue factor) and for the INTEM channel (contact activation) are displayed. No difference was observed for the two treatments (EXTEM: CT *p*-value = 0.3, CFT *p*-value = 0.3, MCF *p*-value = 0.07; INTEM: CT *p*-value = 0.7, CFT *p*-value = 0.5, MCF *p*-value = 0.5).

Coagulation Parameters	EXTEM			INTEM		
	CT (s)	CFT (s)	MCF (mm)	CT (s)	CFT (s)	MCF (mm)
Test 1						
IgG	69	95	61	177	72	60
Anti-EpCAM	74	116	55	207	71	76
Test 2						
IgG	68	162	48	213	139	48
Anti-EpCAM	109	185	44	211	150	47
Test 3						
IgG	67	182	49	186	106	53
Anti-EpCam	71	178	47	175	105	51
Normal range	38–79	34–159	50–72	100–240	30-110	50–72

**Table 3:** Removal of CTCs from blood samples of cancer patients. *n* = number. Samples 1, 2 and 3: from prostate cancer patients. Samples 4 and 6: from pancreatic cancer patients. Sample 5: from a colon cancer patient.

CTC	*n* CTC without Treatment	*n* CTC with Treatment	% CTC Removed
Sample 1	1946	620	68
Sample 2	95	22	77
Sample 3	161	1	99
Sample 4	8	5	37
Sample 5	75	13	83
Sample 6	11	6	45

## Data Availability

Anonymized study data are available from the corresponding author upon reasonable request.

## Supplementary Materials

The following supporting information can be downloaded at https://www.mdpi.com/article/10.3390/pharmaceutics14071397/s1.
