# Supplementary material for: Removal of Circulating Tumor Cells from Blood Samples of Cancer Patients Using Highly Magnetic Nanoparticles: A Translational Research Project
Source: Pharmaceutics. 2022 Jul 1;14(7):1397. doi: 10.3390/pharmaceutics14071397 (PMC9315588; doi:10.3390/pharmaceutics14071397)
Supplement: Supplementary file 1 [file pharmaceutics-14-01397-s001.zip › pharmaceutics-1744141-supplementary.pdf]

Supplementary Materials: Removal of Circulating Tumour Cells from Blood Samples of Cancer Patients Using Highly Magnetic Nanoparticles: A Translational Research Project

Simon Doswald, Antoine F. Herzog, Martin Zeltner, Anja Zabel, Andreas Pregernig, Martin Schläpfer, Alexander Siebenhüner, Wendelin J. Stark and Beatrice Beck-Schimmer

Experimental procedures

General Information

The nanoparticles were analyzed by FTIR spectroscopy (5% in KBr using a Tensor 27 Spectrometer, Bruker Optics equipped with a diffuse reflectance accessory, DiffusIR, Pike Technologies, 200 scans). Elemental analysis (CHNS) was performed with a Vario micro Cube (Elementar).

Synthesis of C/Co-PhEtOH

Following previously described synthesis (1) 10 g carbon-coated cobalt nanoparticles (C/Co) were added to 400 ml of distilled water. The nanoparticles were dispersed using an ultrasonication bath (10 min, Sonorex Digitec DT 103 H, Bandelin). In a 100 ml round-bottom flask, 4-aminophenethyl alcohol (1.2 g, 8.76 mmol) was mixed with 30 ml of distilled water and dissolved by addition of 10 ml hydrochloric acid (HCl conc./37% fuming). The solution of 4-aminophenyl alcohol was added to the dispersed particles before being placed for an additional 5 min in an ultrasonication bath. The nanoparticle dispersion was then placed in an ice bath before a sodium nitrite solution (1.2 g, 17.4 mmol in 10 ml of distilled water) was added dropwise. The reaction vessel was then placed in an ultrasonication bath for 2 h. The nanoparticles were thoroughly washed with distilled water (3 x 100 ml), ethanol (3 x 100 ml) and acetone (3 x 100 ml). At every step, the particles were dispersed in an ultrasonication bath for 3 min and separated by application of a permanent magnet (magnetic decantation). After washing, the particles were dried overnight in a vacuum oven at 50°C.

Synthesis of C/Co-PhEtO$^-$Na$^+$

Following previously described syntheses (2, 3) 0.5 g of C/Co-PhEtOH were added to 20 ml of a sodium methoxide solution (2 M in dry methanol). After being dispersed with an ultrasonic bath for 2 min, the solution was stirred overnight at 65 °C. The particles were washed with dry methanol (8 x 10 ml). At every step, the particles were dispersed in an ultrasonication bath for 3 min and separated by application of a permanent magnet (magnetic decantation). After washing, the particles were dried overnight in a vacuum oven at 50 °C.

Elemental microanalysis

[C] =5.13 %, [H] = 0.24%, [N] = 0.1%, [S] =0%

Synthesis of C/Co@polyglycidin

Following previously described synthesis (4) 0.5 g C/Co-PhEtO$^-$Na$^+$ were added to 20 mL of dry toluene. After being dispersed with an ultrasonic bath for 2 h, the reaction mixture was degassed by bubbling nitrogen through for 30 min. After installation of a reflux condenser, the reaction mixture was heated up to 140 °C under inert conditions. Once the reaction mixture reached 140 °C, 10 ml (+/-)-glycidol (+/- -oxiran-2-ylmethanol) was slowly added with a syringe pump (1.3 ml/h). The reaction mixture was then stirred for 16 h before being down to room temperature. The nanoparticles were thoroughly washed with

toluene (100 ml), methanol (100 ml) and distilled water (100 ml). At every step, the particles were dispersed in an ultrasonication bath for 3 min and separated by application of a permanent magnet (magnetic decantation). The washing process with water was repeated until no foam generation could be observed anymore. After washing, the particles were dried overnight in a vacuum oven at 50 °C.

Elemental microanalysis

[C] = 10.3 %, [H] = 1.2 %, [N] = 0.07 %, [S] = 0 %

ΔC = 5.17 % = 4.31 mmol/g, ΔH = 1.08 % = 9.6 mmol/g, ΔN =-0.03 % = -0.02 mmol/g, ΔS =0 %

Calculated amount of polyglycidol (from difference in Carbon content): 1.44 mmol/g nanoparticles.

This leads to a calculated average chain length: 15 units per starter.

Infrared spectroscopy

Peak list: 2873 cm^{-1}, 2356 cm^{-1}, 1469 cm^{-1}, 1328 cm^{-1}, 1068 cm^{-1}, 923 cm^{-1}, 862 cm^{-1}

Synthesis of C/Co@polyglycidyl-COOH

Following previously described synthesis (5) 300 mg C/Co@polyglycidin were added to 15 ml of dry dimethylformamide. After being dispersed with an ultrasonic bath for 3 min, succinic anhydride (150 mg, 1.3 mmol) was added. The solution was placed for 10 min in an ultrasonication bath before N,N-dimethylpyridin-4-amine (DMAP, 180 mg, 1.5 mmol) and triethylamine (1.5 ml, 10.8 mmol) were added. The reaction mixture was degassed by bubbling nitrogen through for 30 min. The reaction mixture was heated up to 70 °C and stirred overnight under inert conditions. The nanoparticles were thoroughly washed with distilled water (3 x 100 ml). At every step, the particles were dispersed in an ultrasonication bath for 3 min and separated by application of a permanent magnet (magnetic decantation).

Elemental analysis

[C] = 14.3 %, [H] = 1.4 %, [N] = 0.26 %, [S] = 0 %;

ΔC = 4 % = 3.3 mmol/g, ΔH = 0.2 % = 2 mmol/g, ΔN =0.19 % = 0.1 mmol/g, ΔS =0 %

Infrared spectroscopy

Peak list: 2939 cm^{-1}, 2873 cm^{-1}, 2675 cm^{-1}, 2360 cm^{-1}, 2356 cm^{-1}, **1725 cm^{-1}**, 1560 cm^{-1}, 1406 cm^{-1}, 1244 cm^{-1}, 1168 cm^{-1}, 1068 cm^{-1}, 838 cm^{-1}

Synthesis of C/Co@polyglycidyl-COO-EpCAM and C/Co@polyglycidin-COO-IgG

1-Ethyl-3-(3-dimethylaminopropyl)carbodiimide (EDC) and N-hydroxysulfosuccinimide (sulfo-NHS) was dissolved in activation buffer (OceanNanotech) at concentrations of 4 mg/ml and 2 mg/ml, respectively. The solutions were homogenized by vortexing for 10 sec. C/Co@polyglycidyl-COOH nanoparticles solutions (5 mg/ml in activation buffer) were prepared. After being dispersed with an ultrasonic bath for 2 min, 200 µl of the solution were added to 1.5 ml Eppendorf tubes containing 100 µl of activation buffer. The EDC and sulfo-NHS solutions were mixed in a 1:1 volume ratio and 10 µl were added to each nanoparticles-containing Eppendorf tube. To ensure homogeneous dispersion, the solutions were vortexed for 10 sec, followed by 20 sec in an ultrasonication bath. The activation step was done by shaking the Eppendorf tubes at 1200 rpm for 10 min at 25°C in a thermomixer (ThermoMixer Comfort, Eppendorf). 100 µl of an antibody solution (anti-EpCAM or IgG isotype control; 1 mg/ml) were added to the Eppendorf tubes. To ensure homogeneous dispersion, the solutions were vortexed for 10 sec followed by 20 sec in an ultrasonication bath. The Eppendorf tubes were then shaked at 1200 rpm for 4 h at 25°C in a thermomixer. To quench the reaction, 10 µl of quenching buffer (OceanNanotech) were added to each Eppendorf tube. To ensure homogeneous dispersion, the solutions

were vortexed for 10 sec followed by 20 sec in an ultrasonication bath. The quenching reaction was done by shaking the Eppendorf tubes at 1200 rpm for 30 min at 25 °C in the thermomixer. The particles were washed by placing the reaction vessel in a pre-cooled (4 °C) SuperMag separator (OceanNanotech). To ensure complete separation, the magnet was placed in a fridge for 1.5 h before the supernatant was discarded and replaced with 420 μl of fresh cold PBS (pH 7.4, Life Technologies). The nanoparticles were dispersed by vortexing for 10 sec followed by 20 sec in an ultrasonication bath. The Eppendorf tubes were placed back in the fridge and the washing procedure was repeated 3 times. Once the washing process completed, the solutions were aliquoted (30 μl aliquots) and stored at -20 °C until use.

Particle size distribution

An aqueous solution of carbon-coated cobalt nanoparticles (28 nm diameter, NanoAmor) was prepared at a concentration of 5 ppm in Milli-Q water. 1.5 μl of the solution was deposited on carbon-coated copper grids. After overnight drying, the samples were measured on a Tecnai F30 (FEI, 300 kV). The quantification was done for 428 nanoparticles using an image processing program (ImageJ, NIH).

Removal experiments of CTCs

Cell line experiments: see original text.

Removal of CTCs from the blood from healthy subjects spiked with tumour cells: see original text

Analysis of samples using fluorescence-activated cell sorting (FACS)

see original text

Evaluation of a possible effect of nanoparticles on blood cells

see original text

Evaluation of a possible effect of nanoparticles on the coagulation system

Nanomagnets (final concentration of 0.119 mg ml^{-1} were added to citrate blood, allowed to incubate for 2 minutes at room temperature, and were immediately analysed after magnetic nanoparticle removal (n = 3). Three experiments have been performed and detailed results along with the statistical evaluation can be found in table 2 of the original manuscript.

References

1. Zeltner M, Grass RN, Schaetz A, Bubenhofer SB, Luechinger NA, Stark WJ. Stable dispersions of ferromagnetic carbon-coated metal nanoparticles: preparation via surface initiated atom transfer radical polymerization. Journal of Materials Chemistry. 2012;22:12064.
2. Wang S, Zhou Y, Yang S, Ding B. Growing hyperbranched polyglycerols on magnetic nanoparticles to resist nonspecific adsorption of proteins. Colloids and Surfaces B: Biointerfaces. 2008;67:122-6.
3. Khan M, Huck WTS. Hyperbranched Polyglycidol on Si/SiO2 Surfaces via Surface-Initiated Polymerization. Macromolecules. 2003;36:5088-93.

4. Das P, Jana NR. Highly Colloidally Stable Hyperbranched Polyglycerol Grafted Red Fluorescent Silicon Nano-particle as Bioimaging Probe. ACS Applied Materials & Interfaces. 2014;6:4301-9.

5. Li Z, Chau Y. Synthesis of Linear Polyether Polyol Derivatives As New Materials for Bioconjugation. Bioconjugate Chemistry. 2009;20:780-9.
